# Molecular survey of the haemosporidians *Haemoproteus* and *Leucocytozoon* in *Culicoides* (Diptera: Ceratopogonidae) from the Brazilian Amazon

**DOI:** 10.1590/S1984-29612025048

**Published:** 2025-09-29

**Authors:** Daniel Antônio Braga Lee, Israel de Souza Pinto, Paulo Vitor Cadina Arantes, Maria Clara Alves Santarém, Maria Luiza Felippe-Bauer, João Vitor dos Santos Alves da Silva, Rosangela Zacarias Machado, Marcos Rogério André

**Affiliations:** 1 Universidade Estadual Paulista – UNESP, Faculdade de Ciências Agrárias e Veterinárias – FCAV, Departamento de Patologia, Reprodução e Saúde Única, Vector-Borne Bioagents Laboratory – VBBL, Jaboticabal, SP, Brasil; 2 Instituto Federal de Educação, Ciência e Tecnologia do Espírito Santo – IFES, Ibatiba, ES, Brasil; 3 Fundação Oswaldo Cruz – FIOCRUZ, Instituto Oswaldo Cruz – IOC, Laboratório de Coleção de Diptera – LABDIP, Coleção de Ceratopogonídeos – CCER, Rio de Janeiro, RJ, Brasil

**Keywords:** Culicomorpha, Haemosporida, vector-borne diseases, Culicomorpha, Haemosporida, doenças transmitidas por vetores

## Abstract

Haemosporidians belonging to the subgenus *Haemoproteus* (*Parahaemoproteus*) and the genus *Leucocytozoon* rely on dipteran vectors for transmission, with biting midges (*Culicoides* spp.) and black flies (Simuliidae), respectively, playing essential roles in their life cycles. However, little is known about the potential role of *Culicoides* species as vectors for haemosporidians outside Europe and Asia. This study aimed to investigate the presence of *Haemoproteus* spp. and *Leucocytozoon* spp. DNA in *Culicoides* spp. from the Brazilian Amazon. A total of 345 midges (95 *Culicoides foxi,* 218 *Culicoides hylas*, and 32 *Culicoides leopoldoi*) were collected between February 2022 and February 2023 in the Amazon National Park, Pará. Specimens were morphologically identified, and DNA was extracted using the TRIzol technique. PCR targeting the endogenous *cox-1* gene confirmed successful DNA extraction in 86.7% (299/345) of samples. Nested PCR assays targeting the *cytB* gene of *Haemoproteus* spp. and *Leucocytozoon* spp. did not detect DNA in any sample. The absence of detection suggests that the sampled *Culicoides* species are unlikely vectors, possibly due to feeding preferences or ecological limitations. Future studies targeting both DNA and sporozoites in salivary glands of engorged female *Culicoides* spp. are needed to clarify the vectors involved in the transmission of haemosporidians.

## Resumo

Hemosporídeos dos gêneros *Haemoproteus* (*Parahaemoproteus*) e *Leucocytozoon* dependem de vetores dípteros para sua transmissão, sendo os maruins (*Culicoides* spp.) e borrachudos (Simuliidae), respectivamente, fundamentais em seus ciclos de vida. Contudo, o papel de *Culicoides* como vetores de hemosporídeos fora da Europa e Ásia ainda é pouco conhecido. O presente estudo teve como objetivo investigar a presença de DNA de *Haemoproteus* spp. e *Leucocytozoon* spp. em *Culicoides* spp. da Amazônia brasileira. Foram coletados 345 maruins (95 *C. foxi*, 218 *C. hylas* e 32 *C. leopoldoi*) entre Fevereiro de 2022 e Fevereiro de 2023 no Parque Nacional da Amazônia, Pará. Os espécimes foram identificados morfologicamente e DNA foi extraído utilizando o TRIzol. A PCR baseada no gene endógeno *cox-1* confirmou extração bem-sucedida de DNA em 86,7% (299/345) das amostras. Ensaios de nested PCR baseados no gene *cytB* de *Haemoproteus* spp. e *Leucocytozoon* spp. não detectaram DNA em nenhuma amostra. A ausência de detecção sugere que as espécies amostradas são vetores improváveis, possivelmente devido às preferências alimentares ou fatores ecológicos. Estudos futuros visando detectar DNA e esporozoítos em glândulas salivares de fêmeas ingurgitadas são necessários para elucidar os vetores envolvidos na transmissão de hemosporídeos.

## Introduction

The order Haemosporida (Apicomplexa) comprises alveolate intracellular parasites with heteroxenous life cycles, alternating between vertebrate hosts and blood-feeding dipteran vectors (Valkiūnas & Iezhova, 2022, 2023). Three haemosporidian genera are particularly significant for both veterinary and human health: *Leucocytozoon* spp. (Leucocytozoidae), which infects birds; *Haemoproteus* spp. (Haemoproteidae), which can infect birds and reptiles; and *Plasmodium* spp. (Plasmodiidae), which has a broad host range, including birds, reptiles, rodents, non-human primates, and humans (Valkiūnas & Iezhova, 2022, 2023). While *Plasmodium* spp. are transmitted by mosquitoes (Culicidae), *Leucocytozoon* spp. and *Haemoproteus* (*Parahaemoproteus*) spp. rely on black flies (Simuliidae) and biting midges (*Culicoides* spp.) as their vectors, respectively (Valkiūnas & Iezhova, 2022, 2023). These parasites mainly occur in passerines and other bird families worldwide, possibly causing host mortality or morbidity during the acute phase of infection (Valkiūnas & Iezhova, 2022, 2023).

Biting midges of the *Culicoides* Latreille genus (Diptera: Ceratopogonidae) have been confirmed as vectors of protozoa (*Haemoproteus* (*Parahaemoproteus*) spp., *Hepatocystis* spp., *Leucocytozoon* spp. and *Trypanosoma* spp.), nematodes (*Mansonella ozzardi, Mansonella perstans*, *Onchocerca* spp.), and viruses (bluetongue *Orbivirus* and Oropouche *Orthobunyavirus*) (Sunantaraporn et al., 2022; Kyi Soe et al., 2025). Over 1,340 *Culicoides* species are globally distributed and highly adapted to tropical and subtropical environments, especially those with mud and decomposing plant matter, which favor their oviposition and the development of immature life stages (Santarém & Felippe-Bauer, 2024). Thirteen *Culicoides* species have been pointed out as competent vectors (i.e., capable of supporting complete sporogony of the parasites) of *Haemoproteus* (*Parahaemoproteus*) species worldwide (Valkiūnas & Iezhova, 2022; Žiegytė et al., 2021). For instance, *Culicoides impunctuatus* Goetghebuer and *Culicoides nubeculosus* Meigen, highly abundant in Europe, are capable of transmitting 12 and eight *Haemoproteus* (*Parahaemoproteus*) species, respectively (Žiegytė et al., 2021). Although a total of 14 *Culicoides* species have tested PCR-positive for *Haemoproteus (Parahaemoproteus)* parasites globally, it is well established that avian haemosporidians can persist in both competent and non-competent vectors blood-feeding insects for several weeks post-blood meal. These findings highlight the importance of complementing molecular diagnosis with additional methods—such as the detection of sporozoites in the salivary glands—to accurately confirm vector competence in biting midges (Žiegytė et al., 2022). For instance, a recent study conducted in Lithuania by Chagas et al. (2022) detected *Haemoproteus* (*Parahaemoproteus*) DNA in *Culicoides segnis* Campbell and *C. kibunensis* Tokunaga, along with sporozoites in their salivary glands, thereby confirming their competence as vectors in the transmission of *Haemoproteus* spp. On the other hand, among *Leucocytozoon* species, only *L. caulleryi* has been confirmed to be transmitted by a ceratopogonid, namely *Culicoides arakawe* Arakawa, with molecular evidence reported in China (Yu et al., 2000) and Thailand (Kyi Soe et al., 2025). Additionally, several *Leucocytozoon* genotypes have been detected in biting midges from Russia (Platonova et al., 2024) and Thailand (Sunantaraporn et al., 2022).

In Brazil, 151 species of *Culicoides* have been described so far, 123 of which are found in the Brazilian Amazon (Santarém & Felippe-Bauer, 2024). Although no studies have yet established a relationship between *Parahaemoproteus*/*Leucocytozoon* and biting midges in Brazil, *Haemoproteus* (*Parahaemoproteus*) species are widely distributed across communities of passerine birds in the Brazilian Amazon and Cerrado (Fecchio et al., 2018b, 2018c, 2023). On the other hand, a single study has reported the presence of *Leucocytozoon* sp. in passerine birds (blue-crowned manakins: *Lepidothrix coronata*) from the Amazon (Fecchio et al., 2018a). In addition, coinfection with *Leucocytozoon cariamae* and *Haemoproteus pulcher* has been reported in a red-legged seriema (*Cariama cristata*) from the Cerrado biome (Vieira et al., 2023). It was against this background that the present study aimed to molecularly investigate the presence of *Haemoproteus* and *Leucocytozoon* DNA in *Culicoides* biting midges sampled in the Brazilian Amazon Rainforest.

## Material and Methods

### Sampling and morphological identification of *Culicoides* spp.

Monthly collections of Ceratopogonidae were performed from February 2022 to February 2023 in the Amazon National Park, state of Pará, northern Brazil. In each month and location, six CDC light traps were used – three in the ground level (1.5 m above the ground) and three in the canopy layer (15 m above the ground). The traps operated for two consecutive nights each month of sampling (24 h). They ran from 7:00 p.m. to 7:00 a.m. each day. No bait was used, but the CDC traps were connected to a 350 mL container with 70% alcohol to preserve the captured insects. The sampling sites were selected based on pre-existing trails established by ICMBio for the purpose of conducting research and promoting the Amazon National Park. A total of 315 female biting midges belonging to three different species were collected near two trails in the park: 117 biting midges (*C. foxi* Ortiz n=63; *C. hylas* Macfie n=43*; C. leopoldoi* Ortiz n=11) in the Tracoá base (4° 28’ 25.1” S; 56◦ 17’ 32.6” W) and 198 biting midges (*C. foxi* n=32; *C. hylas* n=148*; C. leopoldoi* n=18) in the Uruá base (4° 32’ 40.7” S; 56° 18’ 34.4” W). The specimens were sampled both at ground level (n=254) (*C. foxi* Ortiz n=72; *C. hylas* Macfie n=168*; C. leopoldoi* Ortiz n=14) and at the canopy (n=61) (*C. foxi* Ortiz n=23; *C. hylas* Macfie n=23*; C. leopoldoi* Ortiz n=15). The collected specimens were stored in 30 mL tubes containing ethanol 96 °GL, morphologically identified and stored at -20 °C until DNA extraction. The morphological identification followed the atlas of the wing photographs of Neotropical *Culicoides* and identification key for the hylas species group (Felippe-Bauer et al., 2009). Although most of the female specimens exhibited a burgundy pigment on their abdomens and/or carried visible eggs, the exact number of individuals in these conditions was not recorded, which led to the inclusion of nulliparous females in the analysis.

### DNA extraction and PCR for endogenous *cox-1* gene

DNA was extracted from each individual specimen using Invitrogen TRIzol reagent (Thermo Fisher Scientific). After DNA extraction, the DNA concentration and quality (260/280 ratio) were assessed using a spectrophotometer (Nanodrop, Thermo Fisher Scientific). To verify the presence of potential PCR inhibitors, a conventional PCR assay based on a ~ 710 bp fragment of the endogenous gene cytochrome c oxidase subunit 1 (*cox-1*) of invertebrates was performed (Folmer et al., 1994). For each reaction, *Lutzomyia longipalpis* Lutz and Neiva and *Ctenocephalides felis* Bouché DNA samples were used as positive controls, and ultrapure sterilized water as negative control. Only samples positive in this protocol were used in the PCR assays for haemosporidians.

### PCR assays for haemosporidians

For the molecular detection of haemosporidians, nested PCR (nPCR) assays based on 478 bp (*Leucocytozoon* spp.) and 480 bp (*Haemoproteus* spp.) fragments of the cytochrome b (*cytB*) genic region of haemosporidians (Hellgren et al., 2004) were performed. For each reaction, *Haemoproteus* sp. DNA previously obtained from an Orinoco goose (Werther et al., 2017) and *Leucocytozoon* sp. DNA kindly provided by Dr. Karin Kirchgatter (Laboratório de Bioquímica e Biologia Molecular, Instituto Pasteur, São Paulo, SP, Brazil) were used as positive controls. Ultrapure sterilized water was used as a negative control.

## Results

A total of 268/315 (85%) specimens were positive in the conventional PCR for the endogenous *cox-1* gene (88/95 [92.6%] *C. foxi*; 164/191[85.8%] *C. hylas*; 16/29 [55.1%] *C. leopoldoi*). Out of those, 225/268 (83.9%) were at ground level (*C. foxi* Ortiz n=72; *C. hylas* Macfie n=146*; C. leopoldoi* Ortiz n=7) and 43/268 (16.1%) at the canopy (*C. foxi* Ortiz n=16; *C. hylas* Macfie n=18*; C. leopoldoi* Ortiz n=9). All samples were negative in the PCR protocols for the detection of *Leucocytozoon* spp. and *Haemoproteus* spp.

## Discussion

In this study, the absence of haemosporidian infection in biting midges might be attributed to infected host availability of the sampled specimens. Of the total analyzed samples, 225/268 (83.9%) were collected at ground level, while 43/268 (16.1%) were sampled at canopy. Previous studies conducted in the Brazilian Amazon Rainforest suggest that the primary blood meal sources for *C. leopoldoi* are sloths (*Choloepus didactylus, Choloepus hoffmanni*) and anteaters (*Tamandua tetradactyla*) (Carvalho et al., 2021). Although *C. foxi* and *C. hylas* have been previously associated with a preference for avian hosts, they have also been observed feeding on other animals, including rodents, dogs, and horses (Gusmão et al., 2015). Previous studies conducted in Brazil have shown that these midges tend to feed opportunistically on available hosts rather than adhering to a specific host class (Gusmão et al., 2015). The absence of haemosporidian infections reported herein might be associated with the eclectic feeding behavior of biting midges and a possible higher availability of mammalian hosts rather than infected birds in the studied sites. Unfortunately, we did not assess the number of parous female specimens that were subjected to DNA extractions. Since biting midges acquire haemosporidian parasites through blood feeding on infected avian hosts, analyzing dipteran specimens that have never fed on blood might have contributed to higher number of negative results.

Nonetheless, previous studies performed in Brazil have detected the occurrence of *Haemoproteus* and *Parahaemoproteus* in passerine bird communities from the Brazilian Amazon (Fecchio et al., 2018b, 2018c, 2023). Moreover, studies conducted in other biomes have detected *Haemoproteus (Parahaemoproteus) coatneyi* and a novel lineage of *Haemoproteus (Parahaemoproteus) erythrogravidus* in Passeriformes from the Atlantic Forest (Oliveira et al., 2020), as well as 17 lineages of *Haemoproteus (Parahaemoproteus)* across 11 Passeriformes families distributed throughout the Atlantic Forest, Caatinga, and Cerrado biomes (Lacorte et al., 2013). *Haemoproteus (Parahaemoproteus) syrnii,* a haemosporidian parasite found in over 30 owl species worldwide, has been recently identified through morphological and molecular analyses in 72.2% (39/54) owls belonging to five species from a rehabilitation center in southeastern Brazil (Atlantic Forest biome) (Barino et al., 2021). Altogether, these findings reinforce that epidemiological cycles of *Haemoproteus* (*Haemoproteus*) and *Haemoproteus* (*Parahaemoproteus*) occur in different bird species and biomes across Brazil. While *Haemoproteus* (*Parahaemoproteus*) haemosporidians are likely transmitted by *Culicoides* midges, there is neither molecular nor parasitological evidence on the occurrence of these apicomplexan protozoa in Ceratopogonidae in the country.

Studies conducted in Thailand (Sunantaraporn et al., 2022; Kyi Soe et al., 2025) have detected *Leucocytozoon* sp. DNA in biting midges, suggesting that several *Culicoides* species (*C. guttifer* de Meijere, *C. huffi* Causey, *C. mahasarakhamense* Pramual, Jomkumsing, Piraonapicha and Jumpato, *C. fulvus* Sen and Das Gupta, *C. oxystoma* Kieffer, and *Culicoides* subgenus *Trithecoides*) may play a role as potential vectors for these haemosporidian parasites. Although the vector competence of these *Culicoides* species for *Leucocytozoon* spp. remains unclear, these findings align with the known distribution of *L. caulleryi*, which is transmitted by *C. arakawe* in Southeast Asia. Authors who have reported *Leucocytozoon* sp. in biting midges beyond this region suggest that such occurrence might indicate abortive parasite development in these insects (Platonova et al., 2024). No previous studies in Brazil have reported *Leucocytozoon* spp. DNA in biting midges and black flies until now.

## Conclusion

The absence of haemosporidian DNA in specimens of three *Culicoides* species, namely *C. hylas, C. foxi* and *C. leopoldoi*, sampled in the Brazilian Amazon is likely due to the eclectic feeding behavior of biting midges and a possible higher availability of mammalian hosts rather than infected birds in the studied locations. Additionally, the analysis of nulliparous specimens may have contributed to the negative results. Alternatively, the non-involvement of these three *Culicoides* species in the transmission of *Haemoproteus* (*Parahaemoproteus*) and *Leucocytozoon* cannot be ruled out. Future studies aiming at detecting both haemosporidians DNA and sporozoites in salivary glands of engorged female *Culicoides* spp. across several Brazilian biomes are much needed in order to shed light on the vectors involved in the transmission of *Parahaemoproteus* and *Leucocytozoon* in the country.

## Data Availability

The data that support the findings of this study can be provided by the corresponding author upon request.
